# Effect of Crystallite Size on the Flexibility and Negative Compressibility of Hydrophobic Metal–Organic Frameworks

**DOI:** 10.1021/acs.nanolett.3c02431

**Published:** 2023-11-30

**Authors:** Liam J. W. Johnson, Diego Mirani, Andrea Le Donne, Luis Bartolomé, Eder Amayuelas, Gabriel A. López, Giulia Grancini, Marcus Carter, Andrey A. Yakovenko, Benjamin A. Trump, Simone Meloni, Paweł Zajdel, Yaroslav Grosu

**Affiliations:** † Centre for Cooperative Research on Alternative Energies (CIC energiGUNE), Basque Research and Technology Alliance (BRTA), Vitoria-Gasteiz 01510, Spain; ‡ Department of Physics, Faculty of Science and Technology, University of the Basque Country (UPV/EHU), Barrio Sarriena s/n, Bilbao 48490, Leioa, Spain; ¶ Department of Chemistry and INSTM University of Pavia Via Taramelli 14, Pavia I-27100, Italy; § Dipartimento di Scienze Chimiche e Farmaceutiche (DipSCF), Università degli Studi di Ferrara (Unife), Via Luigi Borsari 46, I-44121 Ferrara, Italy; ∥ Center for Neutron Research, National Institute of Standards and Technology, Gaithersburg, Maryland 20899, United States; ⊥ X-Ray Science Division, Advanced Photon Source, Argonne National Laboratory, Lemont, Illinois 60439, United States; # Institute of Physics, University of Silesia in Katowice, 75 Pulku Piechoty 1, 41-500 Chorzow, Poland; ∇ Department of Chemistry, Institute of Chemistry, University of Silesia, Szkolna 9, 40-006 Katowice, Poland

**Keywords:** Intrusion/Extrusion, Volumetric Negative Compressibility, ZIF-8, Size Effects

## Abstract

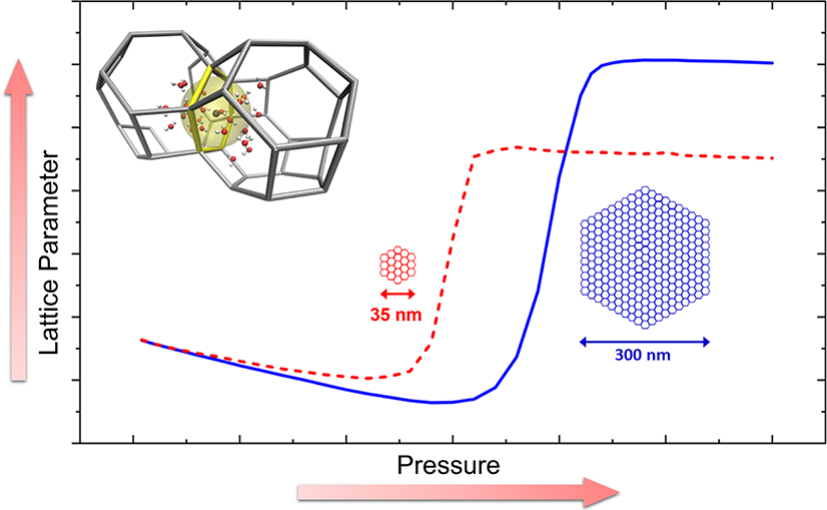

Flexible nanoporous materials are of great interest for applications in many fields such as sensors, catalysis, material separation, and energy storage. Of these, metal–organic frameworks (MOFs) are the most explored thus far. However, tuning their flexibility for a particular application remains challenging. In this work, we explore the effect of the *exogenous* property of crystallite size on the flexibility of the ZIF-8 MOF. By subjecting hydrophobic ZIF-8 to hydrostatic compression with water, the flexibility of its empty framework and the giant negative compressibility it experiences during water intrusion were recorded *via in operando* synchrotron irradiation. It was observed that as the crystallite size is reduced to the nanoscale, both flexibility and the negative compressibility of the framework are reduced by ∼25% and ∼15%, respectively. These results pave the way for *exogenous* tuning of flexibility in MOFs without altering their chemistries.

Flexible materials are of significant interest for use in a wide range of applications, such as sensors,^1,2^ biosensors,^3^ catalysis,^4−7^ material separation,^8,9^ energy storage,^10−15^ anticorrosion coatings,^16^ and water purification.^17^ Of these, metal–organic frameworks (MOFs) are the most explored thus far, with covalent organic frameworks (COFs) and porous organic frameworks (POFs) among others being investigated. MOFs are a type of porous material composed of organic ligands coordinated to metal ions to form 3-dimensional structures. In recent times, they have been extensively investigated for these applications.

MOFs are a relatively recent addition to the family of materials that possess interesting properties, such as increased flexibility and a much greater surface area, in comparison to zeolites and porous silica. These properties make MOFs exciting materials for such applications as absorption and separation as well as for their intrusion/extrusion characteristics. For example, the Zinc Imidazolate Framework (ZIF) ZIF-8 MOF is capable of absorbing molecules larger than the diameter of its pore window due to its high degree of flexibility.^18^ Framework flexibility is a property of MOFs that gives them unique advantages in comparison to other porous media, such as silicas and carbons.

Another key property of the ZIF-8 MOF is negative compressibility (NC).^19^ Negative compressibility is of interest for various applications, including but not limited to actuators,^20^ artificial muscles,^21^ and pressure sensors.^1^ ZIF-8 has even been proposed as a suitable material for smart valves, where volumetric negative compressibility can be used to open/close channels under sufficient pressure.^19^

Materials normally experience contraction during the application of and expansion during the reduction of hydrostatic pressure. However, there are various materials that experience the opposite phenomena when pressure is applied/released. These are classified as materials with a negative compressibility. This can be further subclassified as linear, area, or volumetric NC depending on the number of dimensions in which they demonstrate this characteristic. For example, Yan et al.^22^ have demonstrated that varying the inorganic component of MFM-133(M) (M = Zr, Hf) can alter the linear negative compressibility of the MOF. However, chemical tuning is nontrivial: significant changes in the chemical composition can result in minimal perturbation of macro-scale characteristics, whereas seemingly minor alterations can induce profound changes in these characteristics.

In this Letter, we report the tunable *volumetric* negative compressibility of the hydrophobic MOF ZIF-8 using the *exogenous* property of crystallite size, allowing for the more diverse application of these systems to various technologies. We provide experimental evidence of the effect of crystallite size on the flexibility of ZIF-8 using *in operando* synchrotron experiments applied to intrusion/extrusion cycles of the ZIF-8/water system, reinforcing these experimental findings with molecular dynamics simulations that rationalize the mechanism responsible for this expansion of the system. Moreover, ZIF-8 dispersed in water can also be classified as a Type-III porous liquid,^23,24^ therefore the concepts of giant negative compressibility and flexibility are immediately extended to this class of materials.

Upon the application of hydrostatic pressure to the ZIF-8/water system, the ZIF-8 crystallite experiences normal compression with a linear reduction in lattice parameter with increasing pressure. At a certain value of pressure (dependent on the crystallite size, water is intruded into the material, and the material expands. This expansion correlates with the observed sharp increase in lattice parameter (Figure 1).^25^

**Figure 1:**
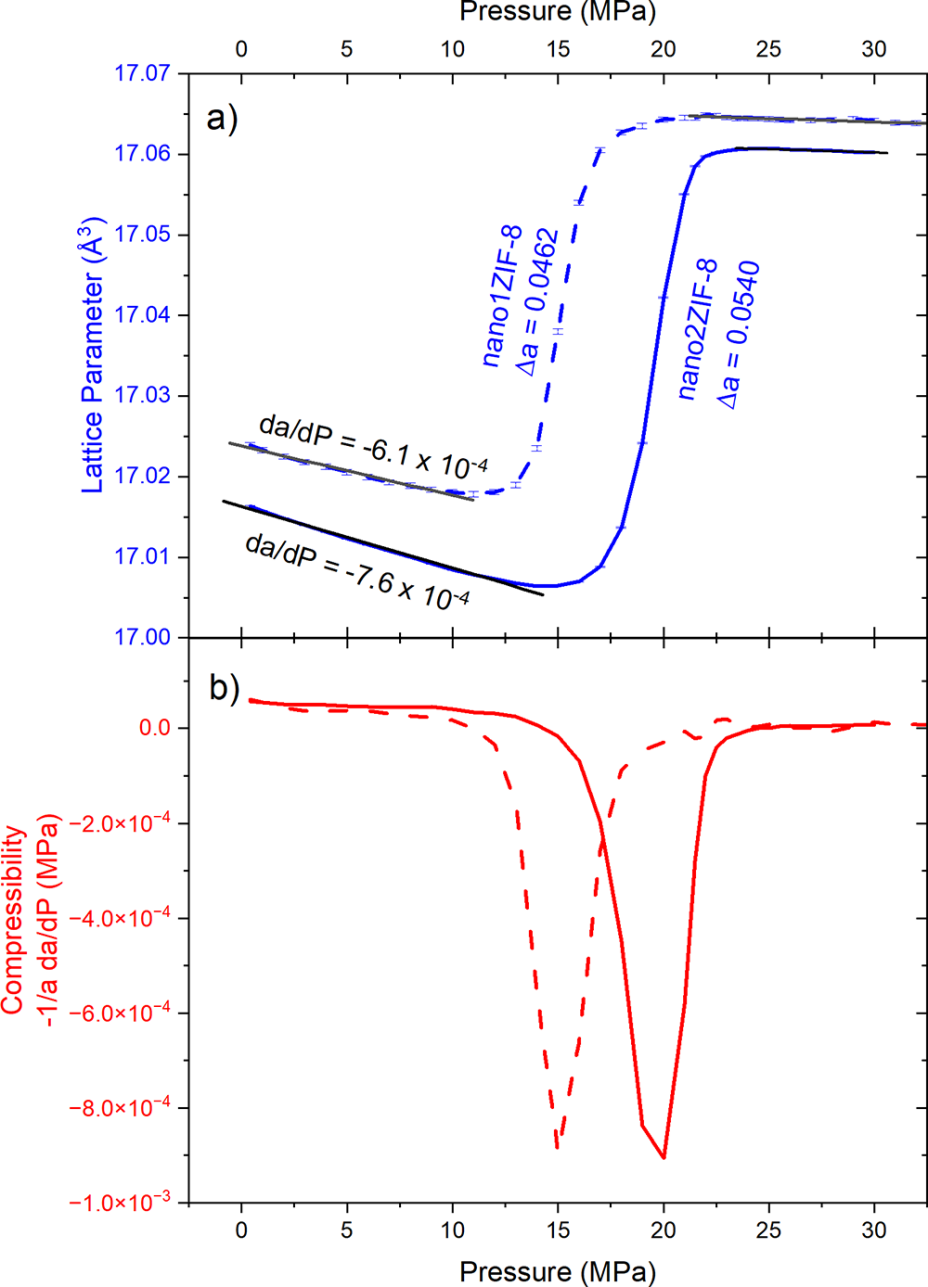
(a) Lattice parameter against applied pressure for nano2ZIF-8 (solid blue line) and nano1ZIF-8 (dashed blue line) systems. Note that the change in lattice parameter with respect to pressure was less for nano1ZIF-8 than it was for nano2ZIF-8. Furthermore,  values differ for the empty cages during compression, suggesting a difference in flexibility between the two samples. (b) Compressibility of nano2/nano1ZIF-8 (solid/dashed) relative to pressure.

Of particular interest here is the effect of the crystallite size on flexibility. As can be ascertained in the linear equations outlined in Figure 1, the preintrusion linear compression for nano1ZIF-8 was ∼25% smaller than that of nano2ZIF-8, suggesting a reduction in material flexibility with a change in crystallite size from 35 to 79 nm. Furthermore, the effect of negative compressibility is ∼16% more pronounced for the sample of nano2ZIF-8 with a larger crystallite size, complementing this observation regarding flexibility. With another increase in crystallite size, a third sample (macroZIF-8) of crystallite size ∼272 nm, measured at 10 °C rather than 5 °C (Table 1), is even more flexible (Figure 2). This variation in temperature has very little effect on the trend of flexibility (Figure S1). The samples with the larger crystallite sizes, referred to as nano2- and macroZIF-8, were acquired from Merck as Basolite Z1200, CAS# 59061–53–9 (Lot: S45328–308) and (Lot: STBG1590 V) respectively and were characterized by XRD (Figure S2, Table S1) and TEM (Figures S3 and S4), whereas the sample of nano1ZIF-8 was synthesized according to a protocol detailed in the Supporting Information, and was characterized by XRD (Figure S2, Table S1), and TEM (Figures S5 and S6).

**Table 1:** Three ZIF-8 Samples, Their Crystallite Size as Established from TEM Images, and Conditions of the Experiments (Temperature and Liquid)

sample name	size (nm)	temperature (°C)	liquid
nano1ZIF-8	35	5	H_2_O
nano2ZIF-8	79	5, 30	H_2_O
macroZIF-8	272	10	D_2_O

**Figure 2:**
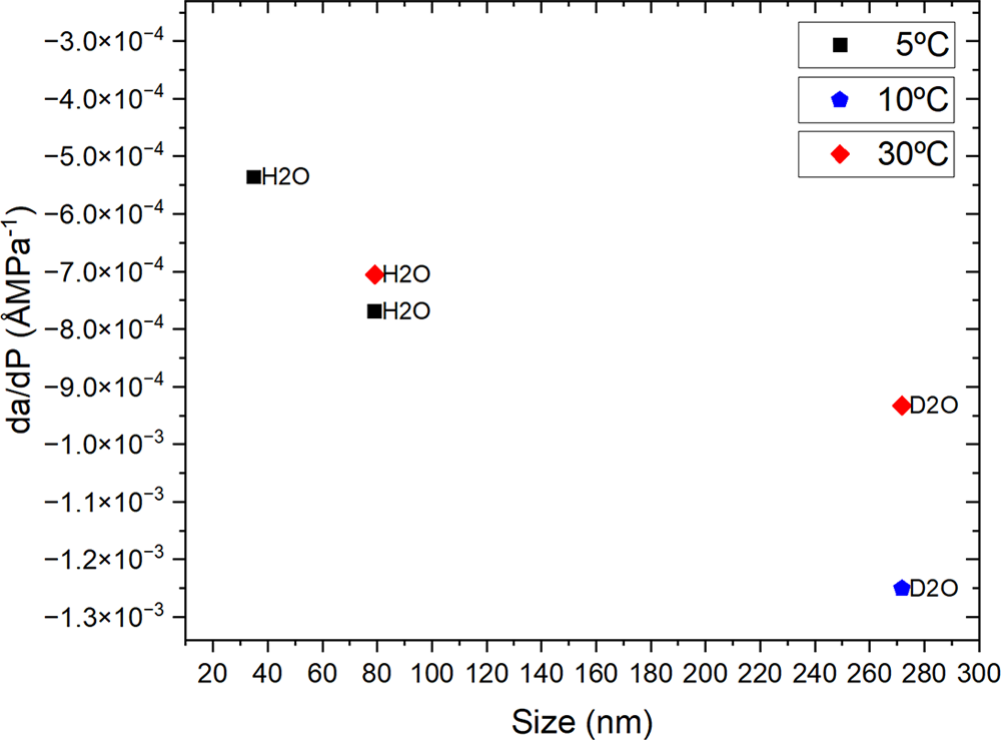
Flexibility vs size for the three systems of ZIF-8 and water/heavy water at different temperatures that are listed in Table 1.

As has been discussed by Tian et al.^26^ and Tanaka et al.,^27^ crystallite size has a significant effect on flexibility, with smaller crystallites having a higher energy penalty for the gate opening mechanism that allows larger gas molecules to be absorbed. The reduced compressibility of nano1ZIF-8 in comparison to the other samples is significant before intrusion. Following from this, one would expect that a higher intrusion pressure (*P*_int_) would be associated with a smaller crystallite size. However, the opposite trend is observed: *P*_int_ grows with increasing crystallite size, with a much greater dependence for crystallites of less than 100 nm.^28,29^ In addition, the third sample, macroZIF-8, extends this trend of crystallite size versus flexibility (Figure 2), which parallels the trend of crystallite size versus *P*_int_ already reported.

The reduction in crystallite size from 79 to 35 nm is associated with a shift in *P*_int_ of approximately 5 MPa at the experimental temperature of 5 °C, correlating well with previous studies^28^ (Figures 1, S8). The ability to tune the extent of, and indeed the trigger pressure for, negative compressibility by *exogenous* properties such as crystallite size is highly desirable as the modification of these properties is far easier than the development of new chemistries for each application. Furthermore, the synthesis of ZIF-8 is straightforward and ZIF-8 MOFs are already commercially available.

To explain the experimental observations at an atomistic level, we performed molecular dynamics (MD) simulations. However, the size of the crystallites considered experimentally (approximately 35, 80, and 270 nm for nano1-, nano2-, and macroZIF-8 respectively) are beyond the scope of atomistic simulations. Therefore, a different approach was taken, in which two different computational samples were considered: the first consists of a slab immersed in water, which has been used successfully in previous works;^28−30^ the second is a triperiodic simulation box obtained by duplicating the unit cell in each direction.^28^ We started our investigation exploring two aspects via the slab sample: (i) whether intruded (potentially metastable) states existed at the experimental *P*_int_ of nano1- and nano2ZIF-8, and (ii) whether the characteristics of the intruded water were different at the different crystallite sizes.

The slab sample was used for the most stable intruded state at 20 and 25 MPa, representative of the different *P*_int_ of nano1- and nano2ZIF-8 crystallites due to the large surface volume ratio of the former (see our recent article^28^). We computed the free energy of the filling of a ZIF-8 cavity positioned in the inner region of the slab (Figure 3), allowing us to minimize possible “surface effects”. The slab geometry is necessary to allow water molecules to move from the bulk water to the interior during the filling of the inner cavity. The computational method to compute the free energy of filling is described in the Supporting Information. One observes that at both 20 and 25 MPa the free energy presents two minima: one at 0 water molecules and another at either 37 or 39 water molecules in the cavity, with the precise position of the minima corresponding to the filled cavity depending on the applied pressure. Interestingly, the increase of pressure of 5 MPa brings an increase of filling of ∼5%. As expected, at 25 MPa the second minimum is deeper, meaning that the stability of the filled state increases with pressure. 37 and 39 water molecules per ZIF-8 cavities were used to investigate the effect of the different *P*_int_ values of the different crystallites on the negative compressibility discussed below.

**Figure 3:**
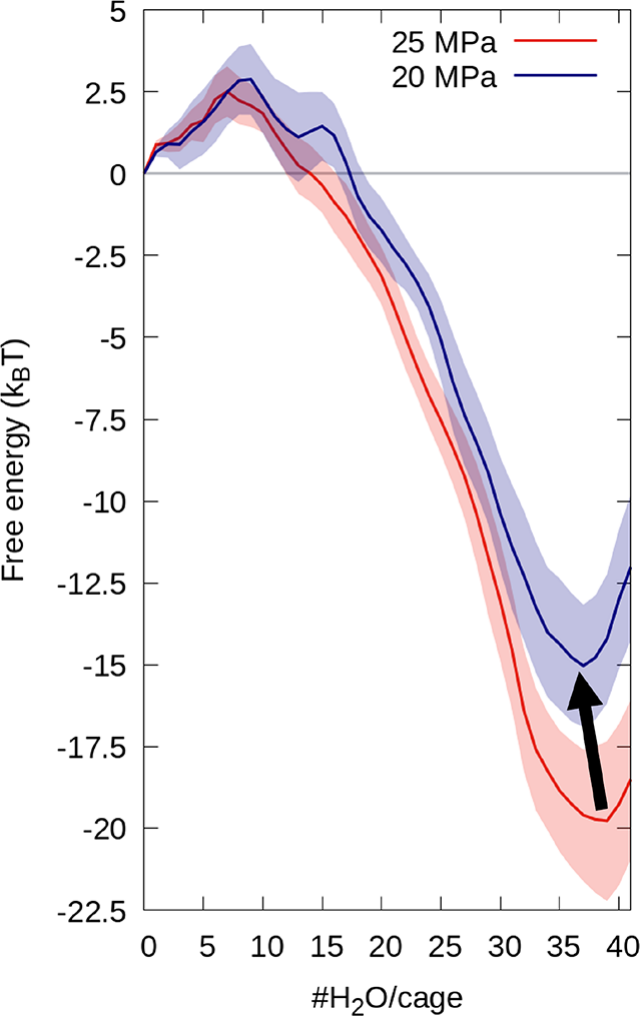
Free energy of filling, expressed in *k*_B_*T*, against the number of water molecules inside a ZIF-8 cage at 20 (blue) and 25 MPa (red). Reducing the pressure, the energy of the stable-state is reduced by ∼5 *k*_B_*T*. Moreover, the minimum is also reached with fewer water molecules, moving from 39 to 37 molecules per cage.

To compute the negative compressibility due to liquid intrusion, we investigated a triperiodic system at 20 and 25 MPa, empty and filled with the number of water molecules corresponding to the minimum of the free energy at the relative pressure. This allowed us to isolate the effect of pressure and water filling on the negative compressibility. We emphasize once again that simulating macro- (∼250 nm) and nanocrystallites (∼50 nm) including bulk water around them is computationally unfeasible, requiring several hundred millions of water molecules. From simulations on the triperiodic system, we computed the average lattice parameter for the four cases: the two pressures, both filled and empty ZIF-8. Given the flexibility of the material, large fluctuations of the lattice parameter were observed during this investigation, which required 70 ns long simulations for each cage to obtain accurate estimates. Additional details on the calculation of the average lattice parameter are reported in the Supporting Information.

In spite of the fact that the force field used in these simulations has not been optimized to model ZIF-8 filled with water, our simulations reproduce several characteristics of the empty and filled states. The fluctuations of the unit cell volume are larger when the cell is empty than when it is filled. Considering the relation between fluctuations and isothermal compressibility in statistical mechanics (*K*_*T*_ = ⟨δ*V*^2^⟩/*k*_B_*T*⟨*V*⟩), consistent with experiments, when ZIF-8 is empty its compressibility is greater. Concerning the negative compressibility at 20 and 25 MPa, mimicking purely the effect of intruding ZIF-8 at the two different pressures relative to macro- and nanocrystallite samples, the variation of the lattice parameter Δ*a* upon wetting is smaller than the experimental values (Δ*a* (20 MPa) = 0.010 Å and Δ*a* (25 MPa)) = 0.016 Å). This is probably due to the force field, which is not optimized to quantitatively reproduce the characteristics of ZIF-8 at high pressures. Nevertheless, the difference of expansion of the lattice parameter upon intrusion at 20 and 25 MPa (ΔΔ*a* = Δ*a* (25 MPa) – Δ*a* (20 MPa)) is 0.006 Å, to be compared with an experimental value of 0.008 Å as obtained from *in operando* diffraction data. Thus, simulations suggest that the effect of the size of crystalline grains on negative compressibility is mainly due to the difference of the *P*_int_ in the nano1- and nano2ZIF-8 samples. The additional effect seen experimentally may come from the greater flexibility of larger crystallites, as can be seen from Figure 2.

As we have shown in a recent article,^28^*P*_int_ can be tuned by changing the crystallite size of ZIF-8, which affects the area/volume ratio of crystallites. In combination with the results presented here, we conclude that crystallite size, through its effect on the *P*_int_ and on flexibility, affects the variation of the lattice parameter and thus can be used to tune the negative compressibility of ZIF-8.

In this work, direct observation of the effect of crystallite size on the flexibility of MOFs has been reported, provided by *in operando* measurements of the lattice parameter during the application of hydrostatic pressure to the ZIF-8/water system. There was a pronounced reduction in flexibility with the reduction in crystallite size between nano1ZIF-8 and the larger samples, reflected in both the isostatic compression of the MOF prior to intrusion and during intrusion, where the change in the lattice parameter was recorded. Furthermore, this work has been complemented with MD simulations that rationalize the observed relationship between the crystallite size and giant negative compressibility (GNC). It is expected that this work will facilitate the development of tunable, pressure-sensitive systems for smart switches/sensors.

## Supporting Information Available

Further details on experiments; a plot showing the dependence of flexibility on temperature; images of the diffraction patterns from the synchrotron; images of the three ZIF-8 samples by TEM; histograms of the size distributions of the three ZIF-8 samples from TEM after sonication, TEM images of nano1ZIF-8 pre-sonication; complete intrusion/extrusion cycles of nano1- and nano2ZIF-8, plotting *P* against Δ*V*; a plot of intrusion pressure vs size for nano1- and nano2ZIF-8; a plot of the lattice parameter against pressure for both empty and filled ZIF-8 (computationally modeled) (PDF)
