# Supplementary material for: Effect of Crystallite Size on the Flexibility and Negative Compressibility of Hydrophobic Metal–Organic Frameworks
Source: Nano Lett. 2023 Nov 30;23(23):10682–6. doi: 10.1021/acs.nanolett.3c02431 (PMC10722533; doi:10.1021/acs.nanolett.3c02431)
Supplement: Supplementary file 1 — nl3c02431_si_001.pdf [file nl3c02431_si_001.pdf]

Supporting Information: The effect of crystallite size on the flexibility and negative compressibility of hydrophobic metal-organic frameworks

Liam J. W. Johnson,[†,‡] Diego Mirani,[¶] Andrea Le Donne,[§] Luis Bartolomé,[†] Eder Amayuelas,[†] Gabriel A. López,[‡] Giulia Grancini,[¶] Marcus Carter,[‖] Andrey A. Yakovenko,[⊥] Benjamin A. Trump,[‖] Simone Meloni,[*,§] Paweł Zajdel,[*,#] and Yaroslav Grosu[*,†,@]

†*Centre for Cooperative Research on Alternative Energies (CIC energiGUNE), Basque Research and Technology Alliance (BRTA), Vitoria-Gasteiz, 01510, Spain*

‡*Department of Physics, Faculty of Science and Technology, University of the Basque Country (UPV/EHU), Barrio Sarriena s/n, Bilbao 48490 Leioa, Spain*

¶*Department of Chemistry and INSTM University of Pavia Via Taramelli 14, Pavia I-27100, Italy*

§*Dipartimento di Scienze Chimiche e Farmaceutiche (DipSCF), Università degli Studi di Ferrara (Unife), Via Luigi Borsari 46, I-44121, Ferrara, Italy*

‖*Center for Neutron Research, National Institute of Standards and Technology, Gaithersburg, Maryland 20899, USA*

⊥*X-Ray Science Division, Advanced Photon Source, Argonne National Laboratory, Lemont, Illinois 60439, USA*

#*Institute of Physics, University of Silesia in Katowice, 75 Pulku Piechoty 1, 41-500 Chorzow, Poland*

@*Department of Chemistry, Institute of Chemistry, University of Silesia, Szkolna 9, 40-006 Katowice, Poland*

E-mail: simone.meloni@unife.it; pawel.zajdel@us.edu.pl; ygrosu@cicenergigune.com

Abstract

Flexible nanoporous materials are of great interest for application in many fields, such as sensors, catalysis, material separation, and energy storage. Of these, metal-organic frameworks (MOFs) are the most explored thus far. However, tuning their flexibility for a particular application remains challenging. In this work, we explore the effect of the *exogenous* property of crystallite size on the flexibility of the ZIF-8 MOF. By subjecting hydrophobic ZIF-8 to hydrostatic compression with water, the flexibility of its empty framework and the giant negative compressibility it experiences during water intrusion were recorded *via in operando* synchrotron irradiation. It was observed that as the crystallite size is reduced to the nanoscale, both flexibility and the negative compressibility of the framework are reduced by ∼25% and ∼15% respectively. These results pave the way for the *exogenous* tuning of flexibility in MOFs without altering their chemistries.

Various ZIF-8 samples were used in this work. The samples with larger crystallite sizes, referred to as nano2- and macroZIF-8, were purchased from Sigma-Aldrich as Basolite Z1200, CAS# 59061-53-9 (Lot: S45328-308) and (Lot: STBG1590V) respectively. The synthesis of the sample with nanoscale crystallite size, henceforth referred to as nano1ZIF-8, was actually a mixture of three nanoZIF-8 samples: nanoZIF-8a, nanoZIF-8b, and nanoZIF-8c, whose syntheses were performed in three different batches using the same reported procedure[S1] but changing the mixing time for each sample as follows.

$Zn(NO_3)_2{\cdot}6H_2O$, 2-methylimidazole and methanol were acquired from Sigma-Aldrich and used as received. To prepare a batch of ZIF-8 nanoparticles, two methanolic precursor solutions of the metal and the ligand were prepared in two different Erlenmeyer flasks: the precursor solution of the metal was prepared by dissolving $Zn(NO_3)_2{\cdot}6H_2O$ 1.467 g in 100 mL methanol (0.0049 M); the precursor solution of the ligand was prepared by dissolving 2-methylimidazole 3.245 g in 100 mL methanol (0.0395 M). The two solutions were separately mixed until complete dissolution of both components had been achieved. The first solution was then rapidly poured into the second one, whilst the latter was stirred continuously. The

obtained solution was vigorously stirred for a further 3 minutes for sample nanoZIF-8a, 5 minutes for sample nanoZIF-8b, and 15 minutes for sample nanoZIF-8c until the solution became cloudy. The cloudy suspension was immediately decanted into four different 50 mL Falcon vials and centrifuged at 150 Hz for 30 minutes. For each vial, the supernatant solution was disposed of, and the precipitated product pellet was washed twice with fresh methanol (first with 60 mL and then with 30 mL) and centrifuged after each wash at 150 Hz for 60 minutes. After being centrifuged once more, the pellet product was left to dry at room temperature and then finely crushed with a mortar to get a homogeneous white powder.

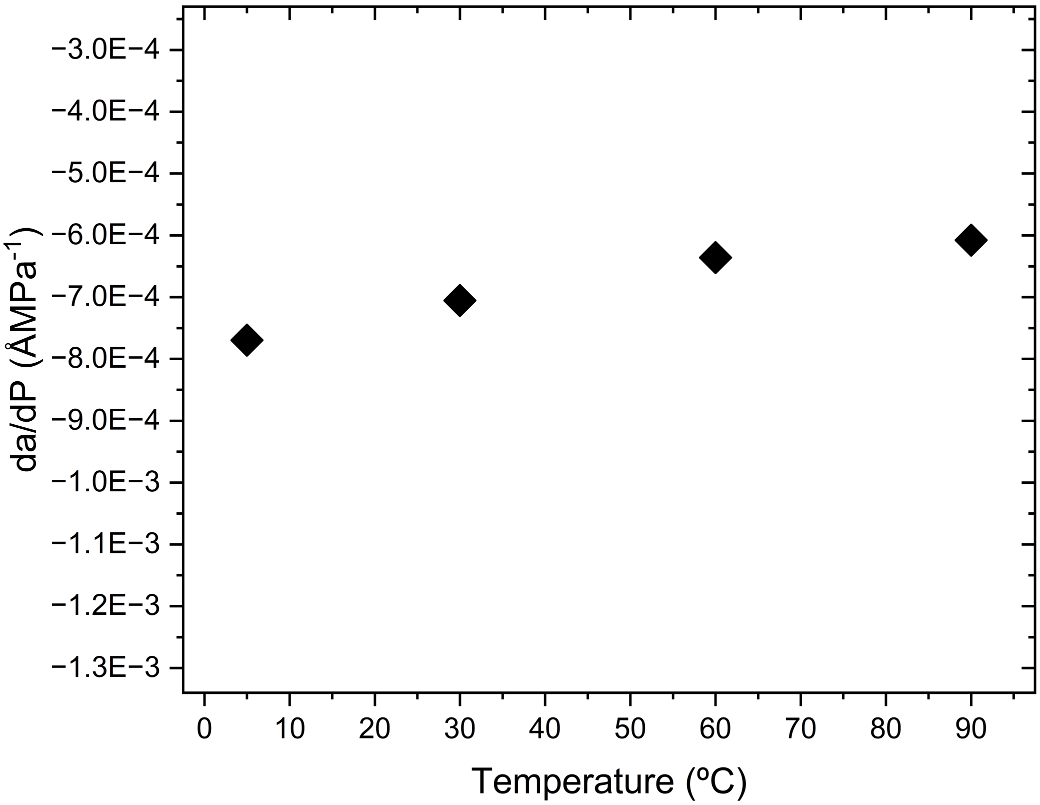

Figure S1: Temperature dependence of flexibility, which is insignificant in comparison to the crystallite size dependence

For the additional data points included in figure 2 of the main text, we have shown that the effect of experimental temperature on flexibility is smaller than that of crystallite size,

and that over the range of temperatures presented in figure 2 of the main text, it is minimal (figure S1).

A Bruker D8 Discover X-ray diffractometer was used with a LYNXEYE-XE detector using CuKα1 radiation ($\lambda = 1.5418$ Å) and Bragg-Brentano θ:2θ geometry. The data collection was conducted at room temperature, between 5° and 40° with a step of 0.02° and a dwell time of 2 s per step. Crystallite size (coherent domain size) was estimated using the Le Bail[S2] method implemented in the Fullprof Suite.[S3] ZIF-8 patterns were generated assuming ideal cubic symmetry with space group I-43m (#217) and laue class m-3m.

The diffraction peaks were visibly broadened in comparison to the standard pattern of Al_2O_3, which was used to measure the machine resolution function. The quantitative analysis of the size broadening was achieved using the spherical harmonics formalism defined by Järvinen.[S4] The model for Laue class m-3m possesses only 5 parameters: K00, K41, K61, K62, K81, from which only the first 2 were used. Possible strain effects were modelled using the parametric approach introduced by Stephens et al.[S5] The refined patterns and the data are presented in figures S2 and table S1 respectively. For the sample named nano1ZIF-8, size analysis (by XRD and TEM) revealed that the sizes of a, b, and c were more or less equivalent, so the three batches were mixed to ensure homogeneity and analysed again with XRD (figure S2a) and TEM (figures S5 - S7).

Table S1: The microstructural parameters refined using the Le Bail method from data acquired at the synchrotron. The average size is given in Ångströms alongside the anisotropy (contained within the braces), as well as the average strain and its associated anisotropy {}, two size parameters, and finally two strain parameters

Sample ID	Lattice Parameter (Å)	Av. Size {anis} (Å)	Strain {anis} (%)
nano1ZIF-8	17.016(2)	422{35}	50{8}
nano2ZIF-8	17.017(2)	1337{1}	18{3}
macroZIF-8	17.047(4)	1086{2}	13{6}

Crystallite size was established using Le Bail fits as established in table S1, figures S2. The samples were imaged by FEI Tecnai F20 transmission electron microscope before and after being dispersed in ethanol and sonicated. The resultant solution post-sonication was

transferred to a carbon mesh fixed on a 3 mm copper grid (200 mesh). TEM images before and after sonication in figures S5 and S6 respectively. TEM images taken after sonication (figures S3-S4, S6) were used to evaluate the crystallite size distribution (figure S7).

To the reviewer and readers:

"Here we have to comment that the "average domain size" based on Scherrer's formula reported in Table S1 can be misleading when comparing both of the nano samples with the macro sample. Putting aside experimental differences between high resolution synchrotron XRD and neutron powder diffraction, one has to take into account actual shape of the distribution of grain sizes as seen by the TEM. The peak broadening formula implemented in Fullprof used to analyze size and microstrain does not take into account possible large dispersions of domain sizes as seen in figure S7 for the macroZIF-8 sample. The refinement is picking up the broadest component, enhancing the "broadest contribution", which comes from the smallest domains. This is not a problem in the case of the monomodally distributed samples nano1 and nano2. The large (over 500%) variance in sizes present for sample macro1 is misrepresented by a small refined coherent domain size. Therefore, to avoid this pitfall, the comparison between samples nano1-, nano2-, and macroZIF-8 is based on the TEM size. Additionally, the general conclusion of grain size dependence holds for nano1 and nano2 both in TEM and XRD sizes, since their size distributions have a similar monomodal shape."

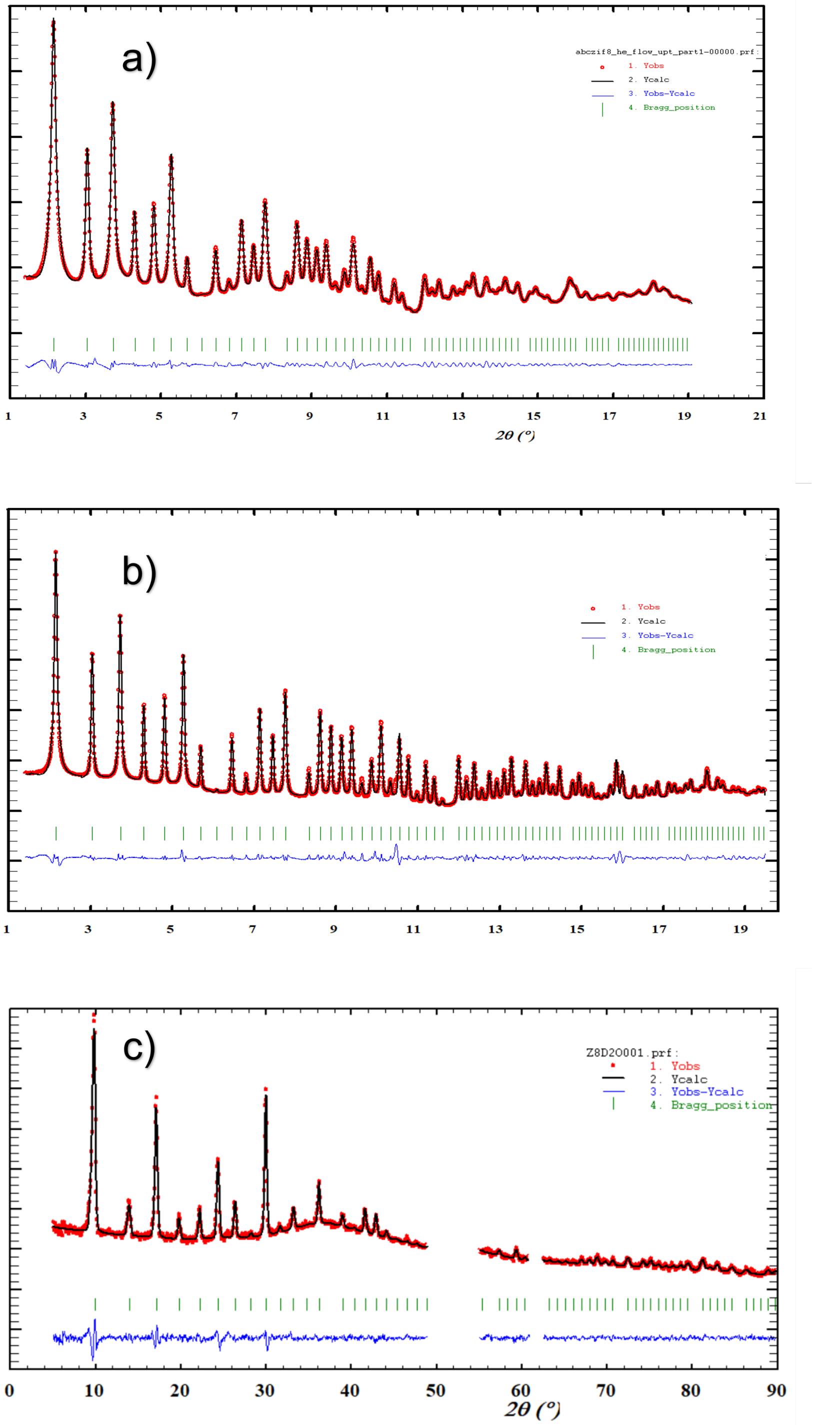

Figure S2: Patterns of a) nano1ZIF-8, b) nano2ZIF-8 were collected at beamline 17BM of the ANL/APS (Lemont, IL, USA) with λ = 0.452 Åand c) macroZIF-8 at BT-1 powder diffractometer instrument at the NCNR (Gaithersburg, MD, USA).

ZIF-8 samples were subjected to water intrusion/extrusion cycling using Pore Master 60 mercury porosimeter from Quantachrome Instruments. macroZIF-8 and nanoZIF-8 samples were mixed with water and encapsulated into flexible, hermetic Teflon capsules prior to testing. High-pressure compression/decompression experiments were performed using an Auto Pore IV 9500 porosimeter (Micromeritics Instrument Corporation, Norcross, USA), where the penetrometer was evacuated to a pressure less than 7 Pa before being filled with mercury to 55 MPa. The system was compressed/decompressed in the required pressure range to observe the intrusion/extrusion phenomena. Each material was evaluated with a minimum of three compression/decompression cycles.

The intrusion/extrusion curves for nano1- and nano2-ZIF-8 are detailed in figure S8. Intrusion volume and intrusion/extrusion pressures detailed in the main text were averages of the values obtained from repeated cycling. The crystallite size effect is evident here, with a reduction of intrusion/extrusion pressures and volume, at lower crystallite size. Furthermore, this difference was in evidence during the *in operando* measurements (figure S9).

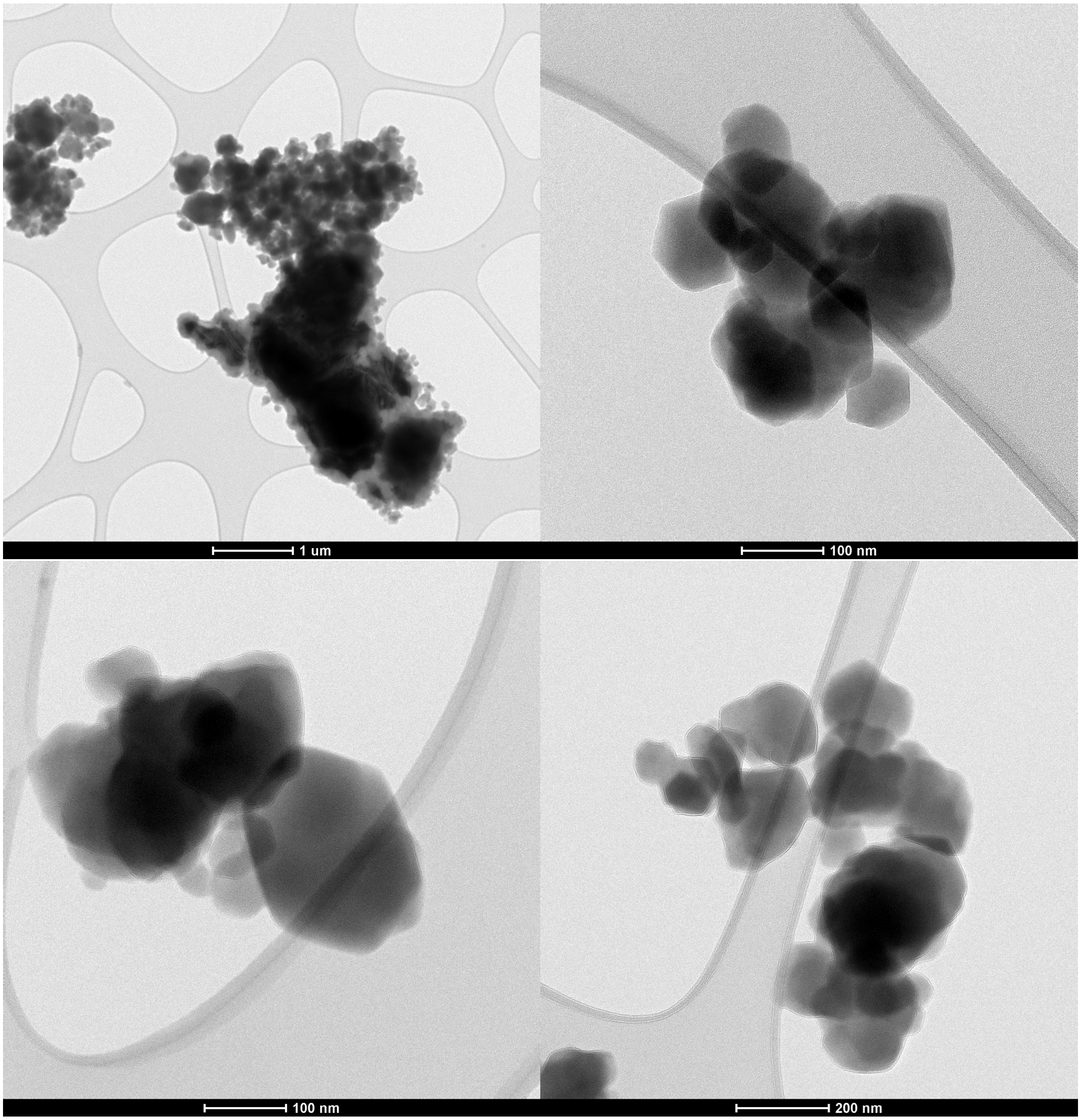

Figure S3: TEM images of nano2ZIF-8 after sonication.

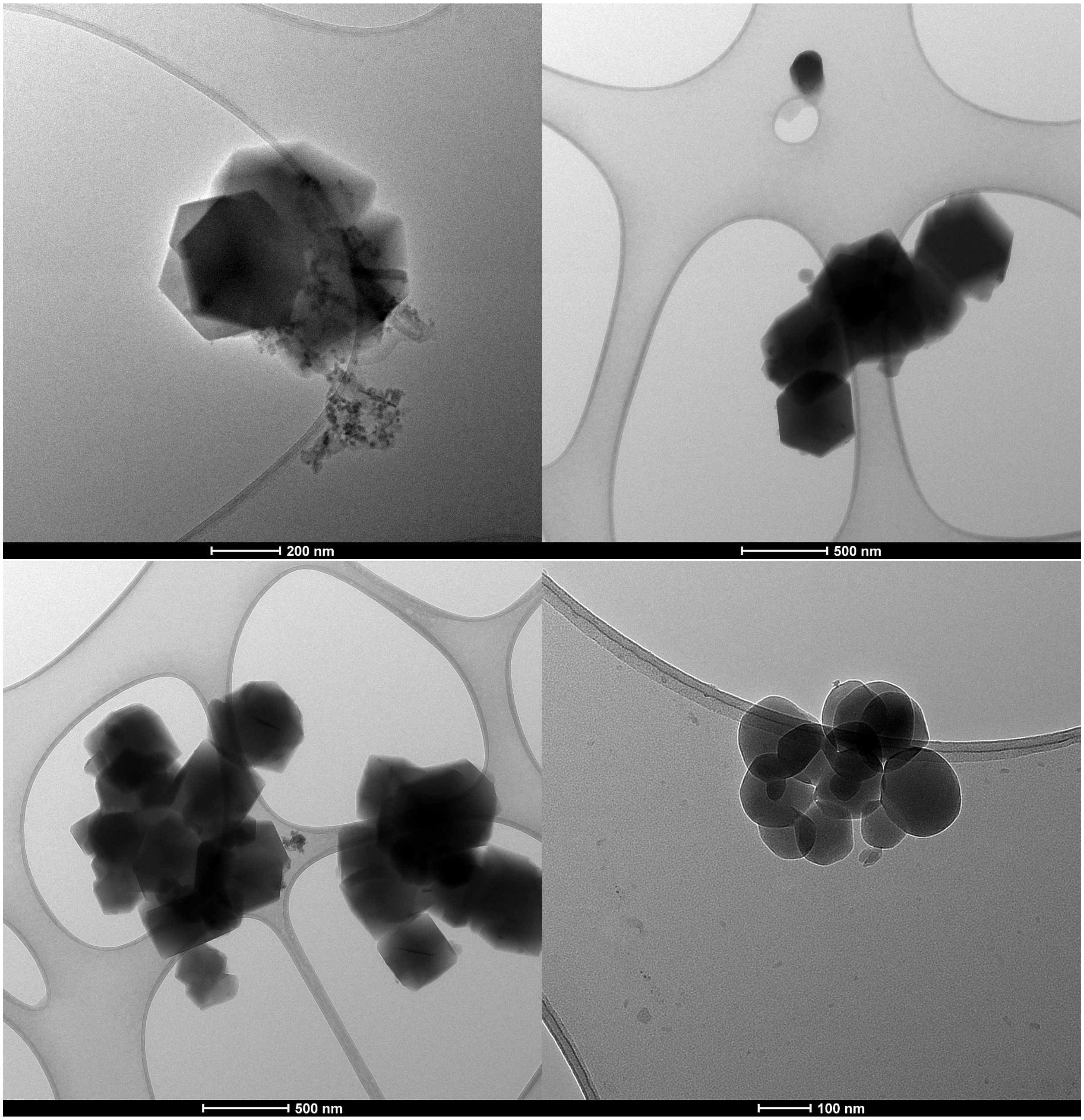

Figure S4: TEM images of macroZIF-8 after sonication.

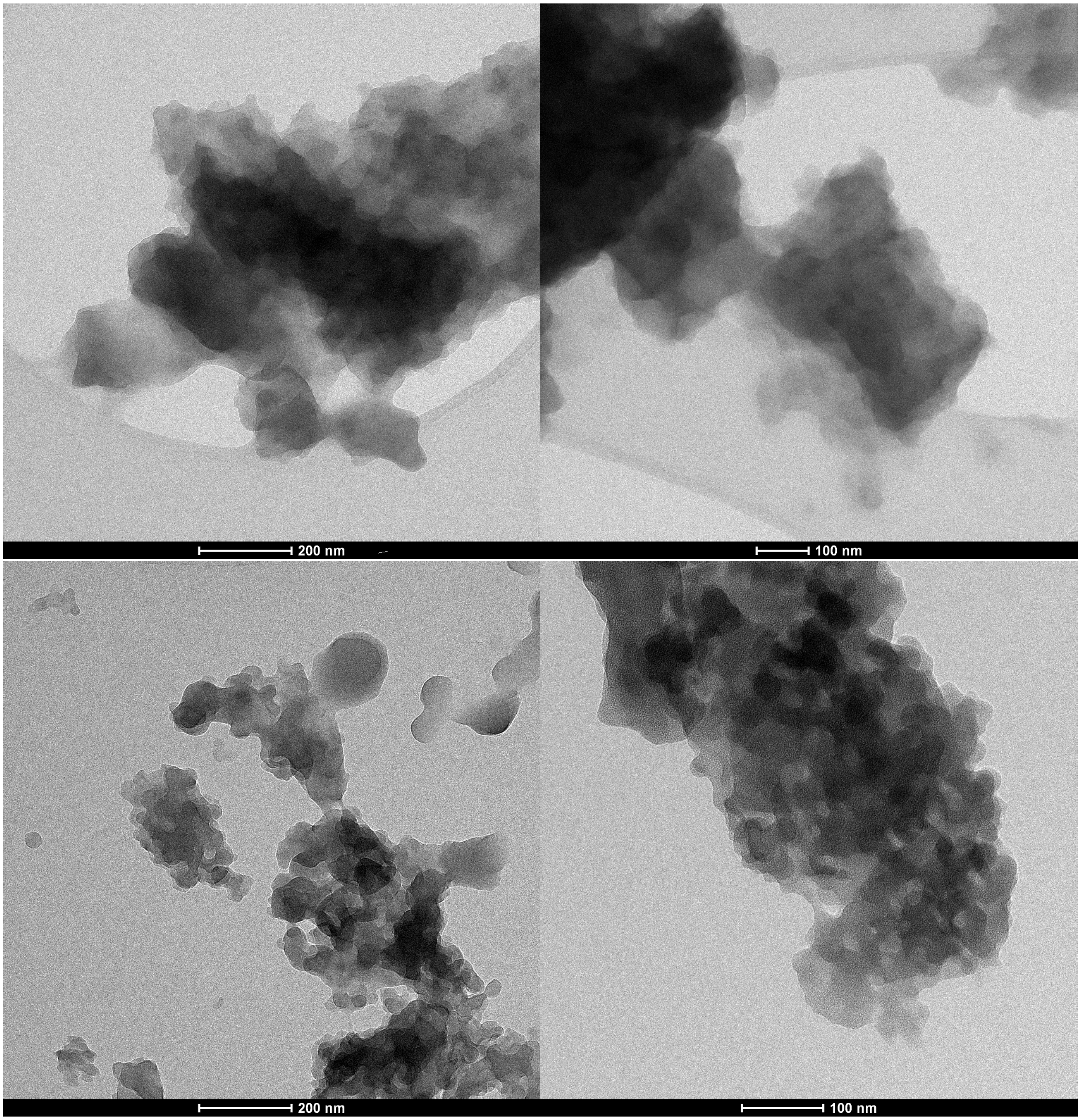

Figure S5: TEM images of nano1ZIF-8 before sonication.

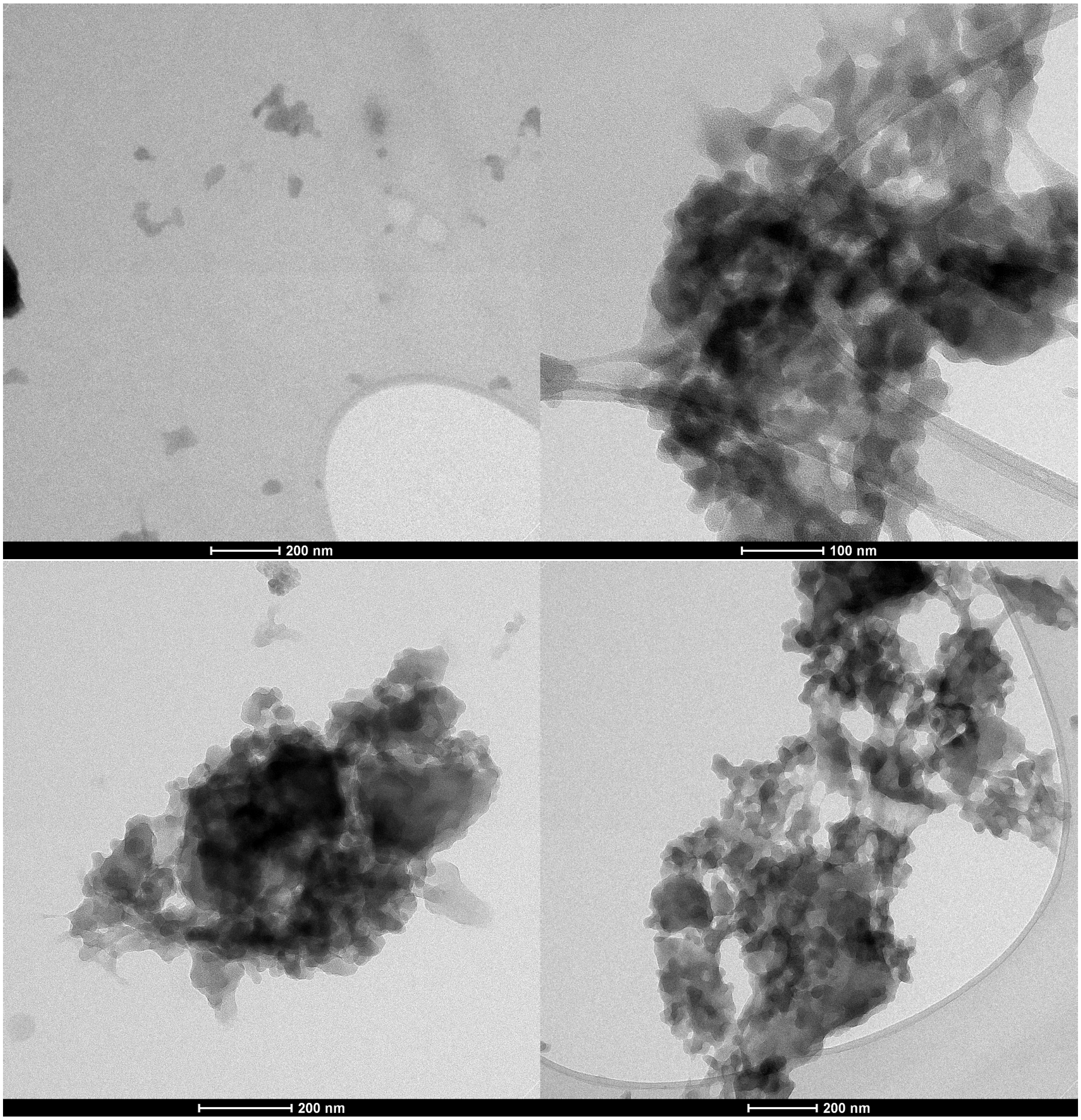

Figure S6: TEM images of nano1ZIF-8 after sonication.

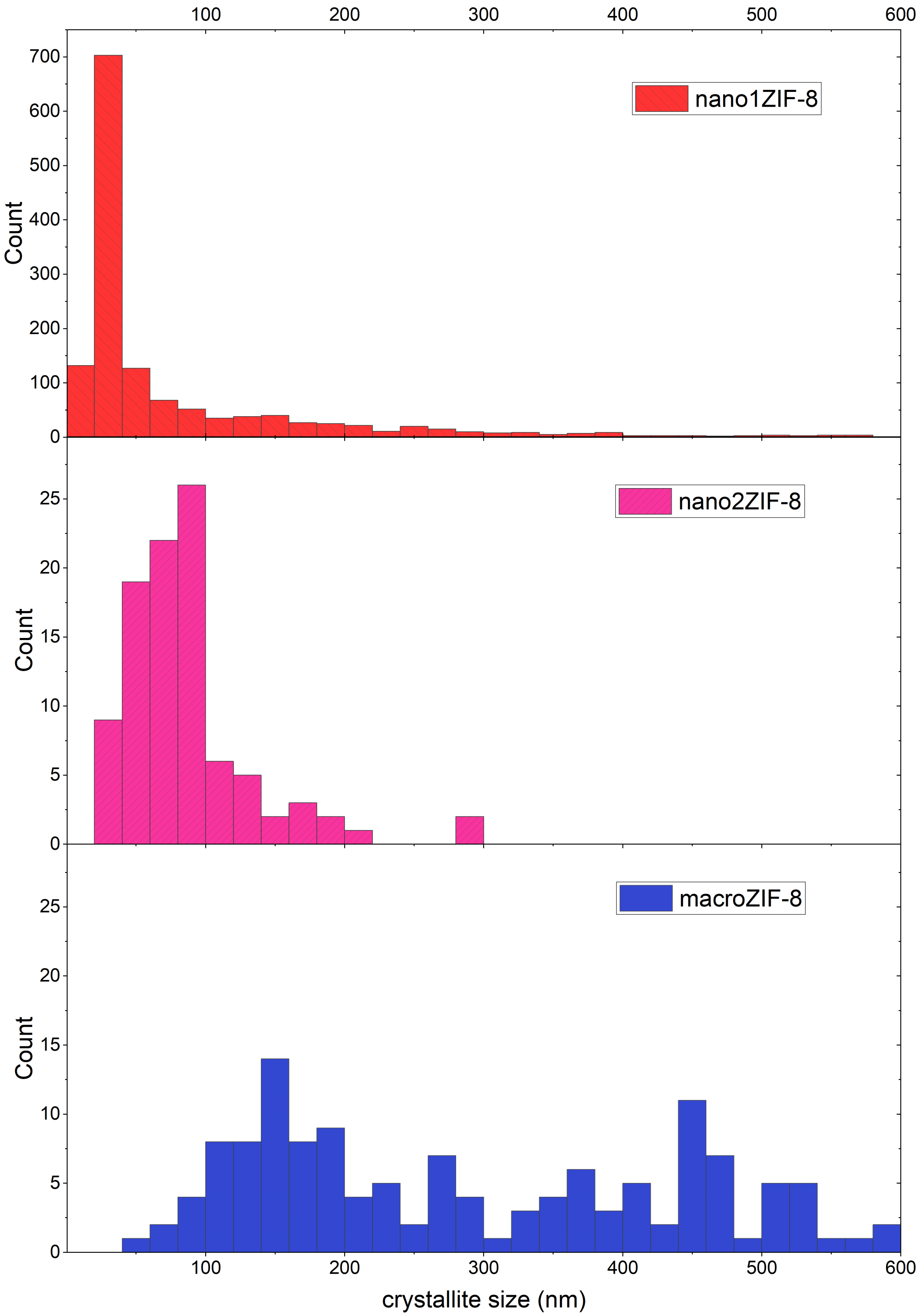

Figure S7: Size distribution of the three ZIF-8 samples measured from TEM images after sonication (figures S4-S6).

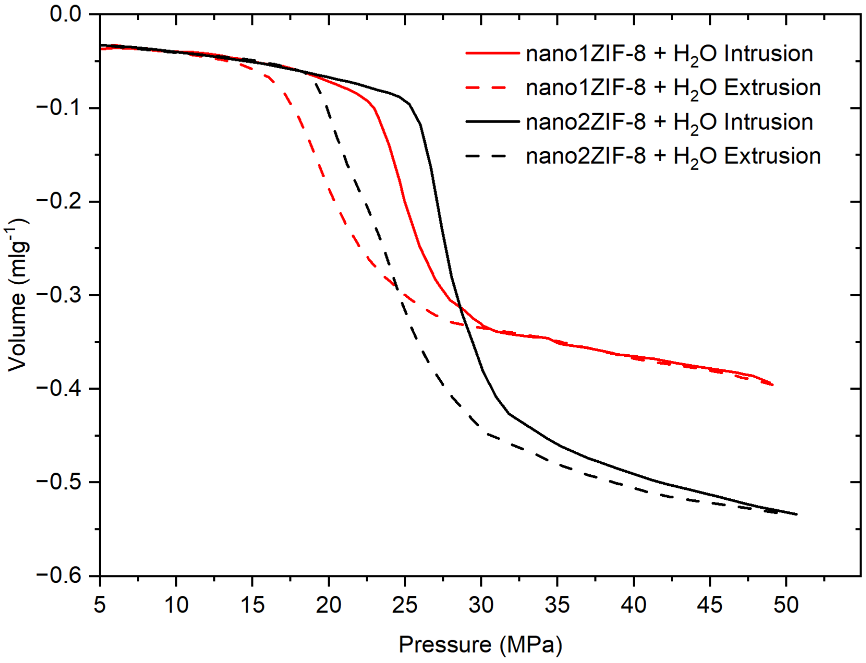

Figure S8: Pressure/volume curves of nano1- and nano2ZIF-8 measured at room temperature.

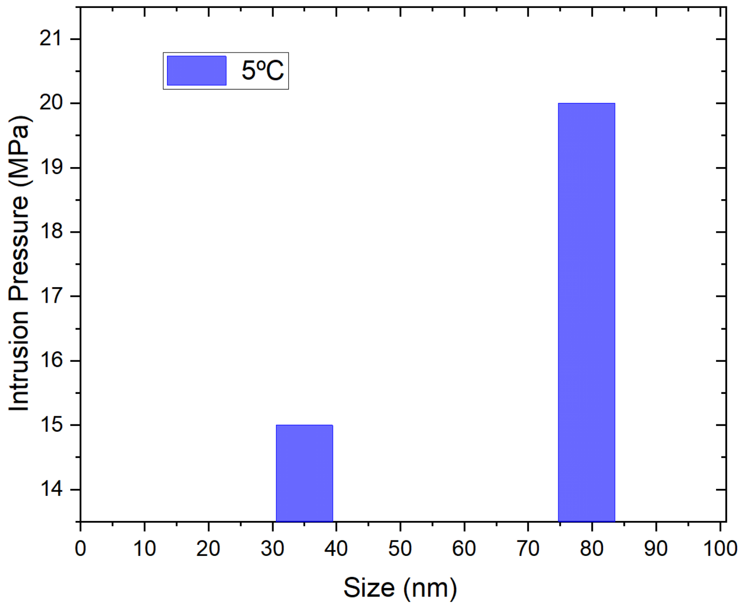

Figure S9: Intrusion pressure dependence on size, as reported in Johnson et al.[S6]

In operando pressure synchrotron powder diffraction studies were carried out at beamline 17BM of the Advanced Photon Source (APS, Lemont, USA).[S7] The wavelength ($\lambda = 0.451810(1)$ Å) and the detector distance (600 mm) were calibrated using NIST SRM660a LaB6. The GSAS-II[S8] and Fullprof[S3] (May 2021) packages were used for pattern processing and refinement respectively. The powdered sample of ZIF-8 was loaded into a sapphire capillary with a K-type thermocouple inserted into the powder but outside the region of the beam. The temperature was maintained using Oxford Cryosystems Cryostream nitrogen blower on the basis of the readout from the thermocouple. Pressure in the system was dynamically stabilized by an ISCO syringe pump which was filled with Water ASTM Type II (VWR Chemicals BDH). The sample was activated *in situ* by flowing He gas at 90°C for 15 minutes. The LeBail analysis was used to extract lattice parameters and estimate effects of peak broadening due to domain size effects and strain induced by the intrusion/extrusion.

Classical Molecular Dynamics simulations were performed using the LAMMPS code,[S9] using the well-established Zheng et al.[S10] force field for ZIF-8 combined with the TIP4P/2005 model of water.[S11] ZIF-8/H_2O interactions were modelled via electrostatics plus the modified $4\epsilon[(\sigma/r)^{12} - c(\sigma/r)^6]$ Lennard-Jones interaction to tune the hydrophobicity of ZIF-8.[S12] The first computational sample consisted of a slab with a thickness of appoximately 19.0 nm (7 unit cells), with a 2 x 2 unit cell extension in the x-y plane (approximately 3.5 x 3.5 nm) and 4800 water molecules, giving a total of approximately 20,000 atoms (ZIF-8 + H_2O). This setup has been successfully used in a number of publications, including Tortora et al.[S13] The second one is a triperiodic supercell box (2 x 2 x 2) filled with 312 (296) water molecules when the pressure applied is 25 (20) Mpa. The volume variation of empty and filled ZIF-8 at the two given pressures is reported in figure S10. The standard error associated to the lattice parameter was estimated as:

$$\sigma_{\bar{a}} = \frac{\sigma}{\sqrt{n}} \quad (1)$$

where σ is standard deviation (obtained by the gaussian fit reported in figure S10) and n is the number of events. It represents the error on the average value given by uncorrelated

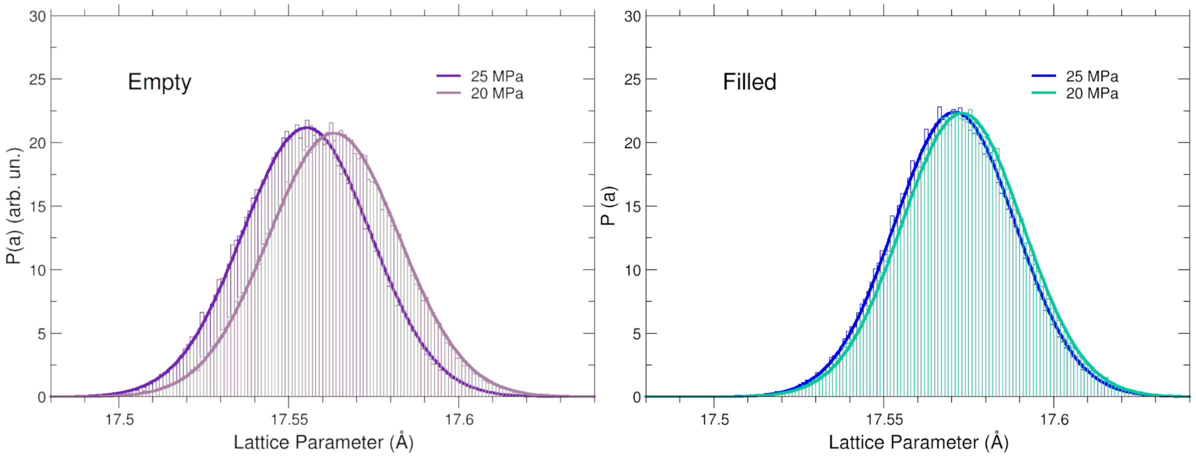

Figure S10: Lattice parameter variation with pressure of empty ZIF-8 (left) and filled ZIF-8 (right). The standard error relative to volume has been calculated following equation 1 and is not visible on the scale of figures. The fact that the trajectory was saved every 500 timestep makes it possible to assume, with good approximation, that the data are uncorrelated.

events.

The simulations were performed within the constant number of particles, pressure, and temperature ensemble denoted as NPT. Here, temperature was controlled via the Nosè-Hoover chains,[S14] while following Marchio et al.,[S15] the pressure in the z-direction orthogonal to the slab was imposed by a mechanical barostat. The x-y in-plane fluctuations of the ZIF-8 slab were allowed according to the Martyna-Tobias-Klein algorithm.[S16]

Restrained Molecular Dynamics (RMD) techniques were applied to compute the free energy of the filling of the ZIF-8 cavity. In statistical mechanics, any thermodynamic potential is related to the logarithm of a suitable probability density function in the relevant ensemble. In the present case, the relevant probability density function is $M(N^*_{\mathrm{H_2O}})$, the probability density that the cage contains a given number of H_2O molecules. In terms of the ensemble distribution $m(\mathbf{r})$, given the probability density to find the atoms of the system in position corresponding to the $6N$ dimensional vector r:

$$M(N^*_{\mathrm{H_2O}}) = \int d\mathbf{r}\ m(\mathbf{r})\ \delta(N_{\mathrm{H_2O}}(\mathbf{r}) - N^*_{\mathrm{H_2O}}) \tag{2}$$

where $\delta(\cdot)$ is the Dirac delta function and $N_{\mathrm{H_2O}}$ is the number of water molecules in the

cavity in the atomistic configuration $\mathbf{r}$. To this probability density is associated the Landau free energy:

$$G(N^*_{\mathrm{H_2O}}) = -k_B T \log M(N^*_{\mathrm{H_2O}}) \tag{3}$$

where $k_B T$ is the thermal energy at the experimental temperature T (k_B is the Boltzmann constant).

To count the number of water molecules in the cage one must first identify the volume of space enclosed in the relevant ZIF-8 cage. We start with a sphere located at the center of the cage and of radius equal to the average distance between the centre and the Zn atoms belonging to the cage. Such a sphere, unlike the true cage, partially intersects the volume of neighbouring cages. Indeed, a sphere sufficiently large to enclose the truncated octahedral cage has a radius equal to $\sqrt{15}/5$ of the average distance between the center of the cage and its Zn atoms. This is the volume we consider in order to count water molecules in the relevant ZIF-8 cavity.

In principle, one can compute $M(N^*_{\mathrm{H_2O}})$ running a long simulation and determining the histogram of $N^*_{\mathrm{H_2O}}$ along it: this histogram is a discrete approximation of $M(N^*_{\mathrm{H_2O}})$, from which one can compute the free energy via Eq. 2. However, it is well known that this approach is computationally inefficient. This is because in the presence of large free energy barriers, larger than the thermal energy $k_B T$, within the typical duration of a simulation (from tens of nanoseconds up to microseconds) the system visits only the free energy well close to the initial configuration.

This problem can be overcome by RMD, which can be considered an extension of constrained MD in order to compute the free energy. In RMD, one introduces a controlled bias forcing the system to explore configurations corresponding to a given number of molecules in the ZIF-8 cage. Consider the Landau free energy of Eq. 3 and, for the sake of simplicity,

assume that the ensemble is canonical, hence:

$$m(\mathbf{r}) = \frac{\exp\left[-\frac{V(\mathbf{r})}{k_B T}\right]}{\int d\mathbf{r}\ \exp\left[-\frac{V(\mathbf{r})}{k_B T}\right]} \tag{4}$$

where $V(\mathbf{r})$ is the physical interacting potential: the force field. Within the canonical ensemble the probability density function of Eq. 2 reads:

$$M(N^*_{\mathrm{H_2O}}) = \frac{\int d\mathbf{r} \exp\left[-\frac{V(\mathbf{r})}{k_B T}\right]\ \delta(N_{\mathrm{H_2O}}(\mathbf{r}) - N^*_{\mathrm{H_2O}})}{\int d\mathbf{r} \exp\left[-\frac{V(\mathbf{r})}{k_B T}\right]} \tag{5}$$

hence, the derivate of the free energy is:

$$\frac{dG(N^*_{\mathrm{H_2O}})}{dN^*_{\mathrm{H_2O}}} = -k_B T \frac{\int d\mathbf{r} \exp\left[-\frac{V(\mathbf{r})}{k_B T}\right]\ \delta(N_{\mathrm{H_2O}}(\mathbf{r}) - N^*_{\mathrm{H_2O}}) \Big/ dN^*_{\mathrm{H_2O}}}{\int d\mathbf{r} \exp\left[-\frac{V(\mathbf{r})}{k_B T}\right]\ \delta(N_{\mathrm{H_2O}}(\mathbf{r}) - N^*_{\mathrm{H_2O}})} \tag{6}$$

$G(N^*_{\mathrm{H_2O}})$ can be computed by the numerical integration of $dG(N^*_{\mathrm{H_2O}})/dN^*_{\mathrm{H_2O}}$. The advantage of this is that the derivative of the free energy can be more easily estimated by atomistic simulations. To achieve this objective, the Dirac delta functions in Eq. 6 are replaced with a smooth Gaussian approximation:

$$\delta\left(N_{\mathrm{H_2O}}(\mathbf{r}) - N^*_{\mathrm{H_2O}}\right) \sim g_\lambda\left(N_{\mathrm{H_2O}}(\mathbf{r}) - N^*_{\mathrm{H_2O}}\right) = \sqrt{2\pi k_B T/\lambda}\ \exp\left[-\frac{\lambda}{2}\left(N_{\mathrm{H_2O}}(\mathbf{r}) - N^*_{\mathrm{H_2O}}\right)^2 \Big/ k_B T\right] \tag{7}$$

Here, $k_B T/\lambda$ is the variance of the Gaussian function, the parameter determining its width and thus the accuracy of the approximation to the corresponding Dirac delta function. The reason for expressing the variance of the Gaussian by the more complex form will become apparent. λ is the only parameter of the RMD method to be computed, and the suitable value can be determined as follows. Within the Gaussian approximation, the derivative of

the free energy, Eq. 6, leads:

$$\frac{dG(N^*_{\mathrm{H_2O}})}{dN^*_{\mathrm{H_2O}}} \sim \frac{\int d\mathbf{r}\ \lambda\left(N_{\mathrm{H_2O}}(\mathbf{r}) - N^*_{\mathrm{H_2O}}\right) \exp\left\{-\left[V(\mathbf{r}) + \frac{\lambda}{2}\left(N_{\mathrm{H_2O}}(\mathbf{r}) - N^*_{\mathrm{H_2O}}\right)^2\right] \Big/ k_bT\right\}}{\int d\mathbf{r}\ \ \exp\left\{-\left[V(\mathbf{r}) + \frac{\lambda}{2}\left(N_{\mathrm{H_2O}}(\mathbf{r}) - N^*_{\mathrm{H_2O}}\right)^2\right] \Big/ k_bT\right\}} \tag{8}$$

Thus, within the Gaussian approximation of the Dirac delta function, the derivative of the free energy can be computed as the expected value of $\lambda\left(N_{\mathrm{H_2O}}(\mathbf{r}) - N^*_{\mathrm{H_2O}}\right)$ over the canonical ensemble of a system driven by the so-called augmented potential $V(\mathbf{r})+\frac{\lambda}{2}\left(N_{\mathrm{H_2O}}(\mathbf{r}) - N^*_{\mathrm{H_2O}}\right)^2$.

In pratice, one computes $\frac{dG(N^*_{\mathrm{H_2O}})}{dN^*_{\mathrm{H_2O}}}$ at the current value of $N^*_{\mathrm{H_2O}}$ by averaging the observable $\lambda\left(N_{\mathrm{H_2O}}(\mathbf{r}) - N^*_{\mathrm{H_2O}}\right)$ along the trajectory of a constant number of particles, constant pressure, and constant temperature molecular dynamics driven by the augmented potential. The operation is repeated for several values of $N^*_{\mathrm{H_2O}}$ in the interval 0 (cage empty) and 42 (cage full of water); then, the so obtained $\frac{dG(N^*_{\mathrm{H_2O}})}{dN^*_{\mathrm{H_2O}}}$ is numerically integrated by the trapezoid rule.

References

(S1) Demessence, A.; Boissière, C.; Grosso, D.; Horcajada, P.; Serre, C.; Férey, G.; Soler-Illia, G. J.; Sanchez, C. Adsorption properties in high optical quality nanoZIF-8 thin films with tunable thickness. *Journal of Materials Chemistry* **2010**, *20*, 7676–7681.

(S2) Le Bail, A.; Duroy, H.; Fourquet, J. L. Ab-initio structure determination of LiSbWO6 by X-ray powder diffraction. *Materials Research Bulletin* **1988**, *23*, 447–452, DOI: 10.1016/0025-5408(88)90019-0, Publisher: Pergamon.

(S3) Rodriguez-Carvajal, J. FullProf for magnetic structures. New features. *Physica B* **1993**, *192*, 55.

(S4) Järvinen, M. Application of symmetrized harmonics expansion to correction of the preferred orientation effect. *Journal of Applied Crystallography* **1993**, *26*, 525–531, DOI: 10.1107/S0021889893001219, Publisher: International Union of Crystallography.

(S5) Stephens, P. W. Phenomenological model of anisotropic peak broadening in powder diffraction. *J. Appl. Cryst* **1999**, *32*, 281–289.

(S6) Johnson, L. J. W.; Paulo, G.; Bartolomé, L.; Amayuelas, E.; Gubbiotti, A.; Mirani, D.; Le Donne, A.; López, G. A.; Grancini, G.; Zajdel, P.; Meloni, S.; Giacomello, A.; Grosu, Y. Optimization of the Wetting-Drying Characteristics of Hydrophobic Metal Organic Frameworks via Crystallite Size: The Role of Hydrogen Bonding between Intruded and Bulk Liquid. *Journal of Colloid and Interface Science* **2023**, *645*, 775–783, DOI: https://doi.org/10.1016/j.jcis.2023.04.059.

(S7) Beamline 17-bm-b: Rapid acquisition powder diffraction. https://www.aps.anl.gov/Beamlines/Directory/Details?beamline_id=88.

(S8) Toby, B. H.; Von Dreele, R. B. GSAS-II: the genesis of a modern open-source all

purpose crystallography software package. *Journal of Applied Crystallography* **2013**, *46*, 544–549.

(S9) Thompson, A. P.; Aktulga, H. M.; Berger, R.; Bolintineanu, D. S.; Brown, W. M.; Crozier, P. S.; J., P.; Kohlmeyer, A.; Moore, S. G.; Nguyen, T. D.; Shan, R.; Stevens, M. J.; Tranchida, J.; Trott, C.; Plimpton, S. J. LAMMPS - a flexible simulation tool for particle-based materials modeling at the atomic, meso, and continuum scales. *Computer Physics Communications* **2022**, *271*, 108171, DOI: https://doi.org/10.1016/j.cpc.2021.108171.

(S10) Zheng, B.; Sant, M.; Demontis, P.; Suffritti, G. B. Force Field for Molecular Dynamics Computations in Flexible ZIF-8 Framework. *The Journal of Physical Chemistry C* **2012**, *116*, 933–938, DOI: 10.1021/jp209463a.

(S11) Abascal, J. L.; Vega, C. A general purpose model for the condensed phases of water: TIP4P/2005. *The Journal of chemical physics* **2005**, *123*, 234505.

(S12) Joly, L.; Ybert, C.; Trizac, E.; Bocquet, L. Hydrodynamics within the electric double layer on slipping surfaces. *Physical review letters* **2004**, *93*, 257805.

(S13) Tortora, M.; Zajdel, P.; Lowe, A. R.; Chorążewski, M.; Leão, J. B.; Jensen, G. V.; Bleuel, M.; Giacomello, A.; Casciola, C. M.; Meloni, S.; Grosu, Y. Giant Negative Compressibility by Liquid Intrusion into Superhydrophobic Flexible Nanoporous Frameworks. *Nano Letters* **2021**, *21*, 2848–2853, DOI: 10.1021/acs.nanolett.0c04941, PMID: 33759533.

(S14) Martyna, G. J.; Klein, M. L.; Tuckerman, M. Nosé–Hoover chains: The canonical ensemble via continuous dynamics. *The Journal of chemical physics* **1992**, *97*, 2635–2643.

(S15) Marchio, S.; Meloni, S.; Giacomello, A.; Valeriani, C.; Casciola, C. Pressure control in

interfacial systems: atomistic simulations of vapor nucleation. *The Journal of chemical physics* **2018**, *148*, 064706.

(S16) Martyna, G. J.; Tobias, D. J.; Klein, M. L. Constant pressure molecular dynamics algorithms. *The Journal of chemical physics* **1994**, *101*, 4177–4189.
